# Balloon-based adjuvant radiotherapy in breast cancer: comparison between ^99m^Tc and HDR ^192^Ir*

**DOI:** 10.1590/0100-3984.2015.0010

**Published:** 2016

**Authors:** Tarcísio Passos Ribeiro de Campos, Carla Flavia de Lima, Ethel Mizrahy Cuperschmid

**Affiliations:** 1 Postdoctoral Fellow, Professor in the Department of Nuclear Engineering, Universidade Federal de Minas Gerais (UFMG), Belo Horizonte, MG, Brazil.; 2 MD, Nuclear Medicine Physician, Doctoral Student in the Graduate Program in Nuclear Sciences and Techniques, Núcleo de Radiações Ionizantes (NRI) at the Universidade Federal de Minas Gerais (UFMG), Belo Horizonte, MG, Brazil.; 3 PhD, Docent at the Center for the History of Medicine, Universidade Federal de Minas Gerais (UFMG), Belo Horizonte, MG, Brazil.

**Keywords:** Breast brachytherapy, Balloon, Radiotherapy dosage, Radiotherapy, adjuvant, ^99m^Tc, ^192^Ir, Monte Carlo method, Braquiterapia de mama, Balão, Reforço de dose, Radioterapia adjuvante, ^99m^Tc, ^192^Ir, Método Monte Carlo

## Abstract

**Objective:**

To perform a comparative dosimetric analysis, based on computer simulations, of temporary balloon implants with ^99m^Tc and balloon brachytherapy with high-dose-rate (HDR) ^192^Ir, as boosts to radiotherapy. We hypothesized that the two techniques would produce equivalent doses under pre-established conditions of activity and exposure time.

**Materials and Methods:**

Simulations of implants with ^99m^Tc-filled and HDR ^192^Ir-filled balloons were performed with the Siscodes/MCNP5, modeling in voxels a magnetic resonance imaging set related to a young female. Spatial dose rate distributions were determined. In the dosimetric analysis of the protocols, the exposure time and the level of activity required were specified.

**Results:**

The ^99m^Tc balloon presented a weighted dose rate in the tumor bed of 0.428 cGy.h^-1^.mCi^-1^ and 0.190 cGyh^-1^.mCi^-1^ at the balloon surface and at 8-10 mm from the surface, respectively, compared with 0.499 and 0.150 cGyh^-1^.mCi^-1^, respectively, for the HDR ^192^Ir balloon. An exposure time of 24 hours was required for the ^99m^Tc balloon to produce a boost of 10.14 Gy with 1.0 Ci, whereas only 24 minutes with 10.0 Ci segments were required for the HDR ^192^Ir balloon to produce a boost of 5.14 Gy at the same reference point, or 10.28 Gy in two 24-minutes fractions.

**Conclusion:**

Temporary ^99m^Tc balloon implantation is an attractive option for adjuvant radiotherapy in breast cancer, because of its availability, economic viability, and similar dosimetry in comparison with the use of HDR ^192^Ir balloon implantation, which is the current standard in clinical practice.

## Abstract (pt)

**Objetivo:**

Análise dosimétrica comparativa entre técnicas de implantes temporários de reforço por meio de balões de ^99m^Tc e de ^192^Ir de alta taxa de dose *(high dose* rate - HDR) mediante simulação computacional. A hipótese é que ambos produzem dosimetria equivalente em condições pré-estabelecidas de atividade e exposição.

**Materiais e Métodos:**

Simulações de implantes com balão preenchido com ^99m^Tc e balão HDR-^192^Ir foram elaboradas no Siscodes/ MCNP5, modelando em voxels um tórax feminino reproduzido de ressonância magnética de mama jovem. Distribuições espaciais de taxas de dose absorvidas foram geradas. Análises dosimétricas dos protocolos foram apresentadas especificando tempo acumulado e atividade requerida.

**Resultados:**

Implante temporário com balão-^99m^Tc apresentou taxa de dose ponderada no leito do tumor, na adjacência do balão, de 0,428 cGyh^-1^.mCi^-1^, e a 8-10 mm distante, de 0,190 cGyh^-1^.mCi^-1^, enquanto o implante de balão com ^192^Ir apresentou 0,499 e 0,150 cGyh^-1^.mCi^-1^, respectivamente. A exposição de 24 horas para balão-^99m^Tc foi necessária para produzir o reforço de 10,14 Gy com 1,0 Ci, ao passo que para balão HDR-^192^Ir foram necessários 24 minutos com segmentos de 10,0 Ci para gerar 5,14 Gy no mesmo ponto de referência, ou 10,28 Gy em duas frações de 24 minutos.

**Conclusão:**

Implante temporário com balão-^99m^Tc é atrativo para a radioterapia adjuvante do câncer de mama, devido a disponibilidade, viabilidade econômica e equivalência radiodosimétrica ao balão HDR-^192^Ir, protocolo presente na prática clínica.

## INTRODUCTION

Worldwide, breast cancer caused 521,000 deaths in 2012^(1)^. In Brazil, the incidence of breast cancer has risen significantly in the last ten years, increasing the number of deaths. The disease is a consequence of genetic factors in combination with social factors, environmental factors, and life habits^(2)^. The factors that favor carcinogenesis and can cause breast cancer include the following^(3,4)^: exposure to carcinogens, such as chemical products and ionizing radiation; parasitic and viral infections; inherited genetic mutations; hormonal and metabolic changes; and immunodeficiency.

Treatment involves surgery, locoregional radiotherapy, chemotherapy, and hormone therapy for systemic control^(5,6)^. Surgical resection margins are defined as a broad margin of possible cancer cell infiltration. The surgical procedure can be conservative or radical^(7)^. Non-conservative surgery involves subcutaneous glandular mastectomy or subcutaneous mastectomy, preserving only the skin and the nipple-areola complex. In contrast, simple or total mastectomy involves removal of the breast including the skin and nipple-areola complex. Modified radical surgery involves mastectomy with partial preservation of the chest muscles and axillary lymphadenectomy, whereas radical surgery involves the complete removal of the chest muscles with axillary lymphadenectomy. Breast conserving surgery includes excision of the tumor with no margin (lumpectomy) or with a margin (segmentectomy or segmental resection). Tumors smaller than 2.0 cm with tumor-free surgical margins can be treated with segmental resection followed by complementary radiotherapy. However, for tumors smaller than 4 cm with tumor-free resection margins, up to 10% of cases treated with conservative treatment involving radiotherapy have been reported to show local recurrence^(8-10)^, leading to psychological trauma and having negative repercussions for the cancer prognosis. Local recurrence depends on the level of aggressiveness and diameter of the tumor, as well as on microscopic involvement of the margins. In breast conserving surgery, complementary (boost) radiotherapy in the tumor area is recommended^(8,11,12)^.

Regardless of the histological type of the tumor, age of the patient, chemotherapy, or hormone therapy, and even with surgical resection margins free of cancer cells, breast conserving surgery is followed by breast irradiation^(7)^. A radiotherapy boost in the area is indicated when patients are below 50 years of age, specimens show more than 25% ductal carcinoma in situ, exiguous margins are less than 1 cm (whether free of cancer cells or not), and the tumor shows a high degree of local aggressiveness^(8-10)^. Postoperative radiotherapy is necessary when treating in situ ductal carcinoma with breast conserving surgery^(8,10)^. Such surgery is considered the standard for the initial stages (I and II). A meta-analysis of clinical trials of breast cancer in the initial stages, conducted by the Early Breast Cancer Trialists' Collaborative Group, revealed the importance of radiotherapy after lumpectomy, showing that irradiation reduced the five-year local recurrence rate from 26% to 7%^(13)^. The metaanalysis also suggested that, for every four local recurrences, one death could be avoided^(14-16)^.

Radiotherapy has evolved in the sense that it offers lower morbidity and greater efficiency in localized control. The treatment arsenal includes partial radiotherapy, intraoperative radiation therapy, the balloon technique, and intensity modulated radiation therapy. High-dose-rate (HDR) ^192^Ir brachytherapy with a balloon implant is frequently applied in partial radiotherapy, using the MammoSite^®^ Radiation Therapy System. The MammoSite system consists of a catheter attached to an inflatable balloon measuring 4-6 cm in diameter, with a ^192^Ir source that provides, for example, 34 Gy in two daily fractions of 3.4 Gy each over five consecutive days^(17,18)^. The balloon may be inserted in the surgical cavity during or after the breast conserving procedure. The balloons come in two sizes, 4-5 cm and 5-6 cm in diameter, and are inflated with saline solution. The balloon should be carefully adapted to the surgical cavity, considering the distance to the skin surface to ensure adequate coverage and dose homogeneity, as well as to lower the risk of complications^(18)^.

The temporary interstitial implant technique with a ^99m^Tc-filled balloon, supplied with ^99^Mo/^99m^Tc generators, was proposed by the Núcleo de Radiações Ionizantes (NRI, Center for the Study of Ionizing Radiation) at the Universidade Federal de Minas Gerais (UFMG, Federal University of Minas Gerais). This technique is justified by the availability of soluble ^99m^Tc supplied with 2.0 Ci generators, for example. Elution can generate 2.0-3.0 mL of an aqueous solution of sodium pertechnetate [Na(TcO_4_)^-^], with sufficient activity for treatment. Furthermore, it allows for successive fractional applications until the established reference doses are achieved. This procedure may be associated with the breast conserving surgery with exposure taking place in the postoperative phase, before radiotherapy. Studies with ^99m^Tc balloons were carried out together with the NRI brachytherapy research using ^166^Ho radioactive seed implants^(19-24)^.

A three-dimensional simulation of nuclear particle transportation aims to eliminate the deficiencies in two-dimensional planning by means of an analytical method in homogeneous medium, and is an important tool in improving the quality of radiotherapy procedures in oncology^(25-27)^. Computational methods have been highly relevant for dosimetric evaluation using heterogeneous models^(26,27)^. The Siscodes (a computational system for neutron/photon dosimetry based on stochastic methods) is a tool used for the construction of computational models and simulation in radiotherapy using stochastic codes, such as the Monte Carlo N-Particle Code (MCNP)^(27-29)^. This system allows the conversion of computed tomography images to a voxel model. Using a database with chemical composition of tissues and nuclear data, the Siscodes associates nuclear and chemical data to the model voxels, by selecting the tissue of each voxel, as well as positioning the teletherapy and brachytherapy sources. The system uses the MCNP for simulating nuclear particle transport in the model. The resulting dosimetric data are presented in the form of spatial distribution of doses and dose-volume histograms^(27)^.

Dosimetric intercomparison is possible when similar exposure conditions are used. This objective of this article was to create such conditions, using computational methods, in order to compare and analyze two protocols-a preclinical protocol, without clinical experimentation; and a clinical practice protocol-a comparison that could not be reproduced in real-life clinical situations. This was a dosimetric inter-comparison of HDR ^192^Ir-filled balloons and ^99m^Tc-filled balloons in temporary breast implants for brachytherapy, simulated under similar conditions and generating dose rate spatial distributions normalized by the source activity and accumulated dose at equivalent reference points. The hypothesis of this study was that both protocols would produce equivalent doses under pre-established conditions of activity and exposure.

## MATERIALS AND METHODS

### HDR **^192^** Ir-filled balloon protocol

A MammoSite-type applicator and centered catheter, located in the upper outer quadrant of the breast, was simulated. The study considered a 4 cm-diameter saline-filled balloon and a 5 mm-long, 1 mm-diameter metallic cylindrical linear segment, filled with ^192^Ir, in the (1,0,0) direction.

### **^99m^** Tc-filled balloon protocol

A ^99m^Tc-filled balloon implant was simulated. The source was defined as a 1.6 cm-diameter sphere, with centers coinciding with a balloon located 3.0 cm from the skin. The emissions were considered uniformly distributed throughout the volume of the balloon.

## Radioactive sources

The characteristics of the nuclear emissions of the radionuclides ^192^Ir and ^99m^Tc, in terms of activity and emission percentages, were adopted according to the Evaluated Nuclear Structure Data Files of the Medical Internal Radiation Dose Committee^(30-32)^.

### Simulator

Twenty-three sequential magnetic resonance imaging (MRI) scans (morphology phase) of the breast of a young patient were selected, comprising 146 slices of 4 mm each. The images were digitalized and combined to form a gray-scale voxel data model. With the Siscodes^(27-29)^, a 2 × 2 × 2 mm 3 voxel model of the breast was created. The anatomical structures were identified in each of the 23 planes, creating a three-dimensional voxel structure. From each image plane, a two-dimensional matrix was created in shades of gray, and the various matrices were combined to form the three-dimensional voxel model. One voxel, volumetrically equivalent to a tissue and identified by a specific color, was associated with each cubic element of the matrix. The chemical composition and density of the tissues were in accordance with the ICRU-44^(33)^.

### Computational codes

The Siscodes was used in order to generate a file containing all of the information of the computational model, in a format accepted by the MCNP5 program (version 5.2). The energy deposition in the voxels was evaluated in MeV.g^-1^) per transition (t). The energy deposition units (MeV.g^-1^.t^-1)^ were transformed into absorbed dose rates based on the activity (cGy.h^-1^.mCi^-1^) present in the balloon (^99m^Tc) or in the source segment (^192^Ir), with a conversion factor of 2133.86. After simulations, the results were incorporated into the Siscodes, generating the spatial distributions of dose. The initial seed activity was calculated in order to generate the target dose in the tumor bed.

### Uncertainties

The computational uncertainties were analyzed for each voxel by using the MCNP5, depending on the number of executed particles. The uncertainties were less than 5% in the breast tissue and were even lower in the voxels closer to the radiation source.

## RESULTS

The model was produced from 23 MRI slices of the mammary gland. A 21 × 60 × 23 element matrix, totaling 28,980 cubic elements or voxels, was generated. The tissue voxel model allowed a three-dimensional structure of the breast to be designed. The chemical composition of the tissues is shown in Table 1.

**Table 1:** Chemical constitutions of tissues and air in a weight-proportional fraction.

Elements	Skin	Adipose	Gland	Muscle	Rib	Lung	Air
Hydrogen	10.0	11.4	10.6	10.2	6.4	10.3	—
Oxygen	64.5	27.8	52.7	71.0	43.6	74.9	76.7
Nitrogen	4.2	0.7	3.0	3.4	3.9	3.1	0.1
Carbon	20.4	59.8	33.2	14.3	26.3	10.5	-
Sodium	0.2	0.1	0.1	0.1	0.1	0.2	-
Potassium	0.1	-	-	0.4	0.1	0.2	—
Phosphorus	0.1	-	0.1	0.2	6.0	0.2	-
Magnesium	—	—	—	—	0.1	—	—
Sulphur	0.2	0.1	0.2	0.3	0.3	0.3	-
Chlorine	0.3	0.1	0.1	0.1	0.1	0.3	-
Density (g.cm^–3^)	1.09	0.86	1.02	1.04	1.92	0.26	0.001

**Figure 1:**
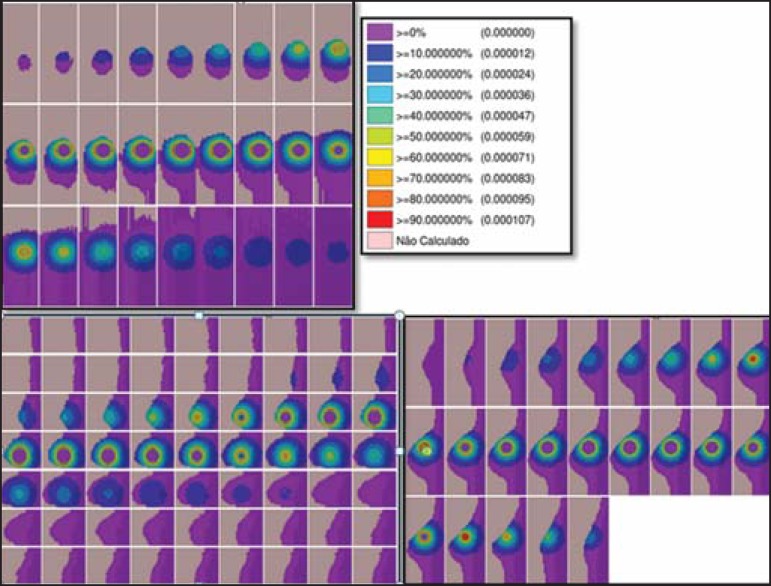
Spatial distribution of normalized dose rate in relation to the activity, shown in lateral, sagittal, and axial slices, induced by brachytherapy with a ^192^Irfilled balloon.

**Figure 2:**
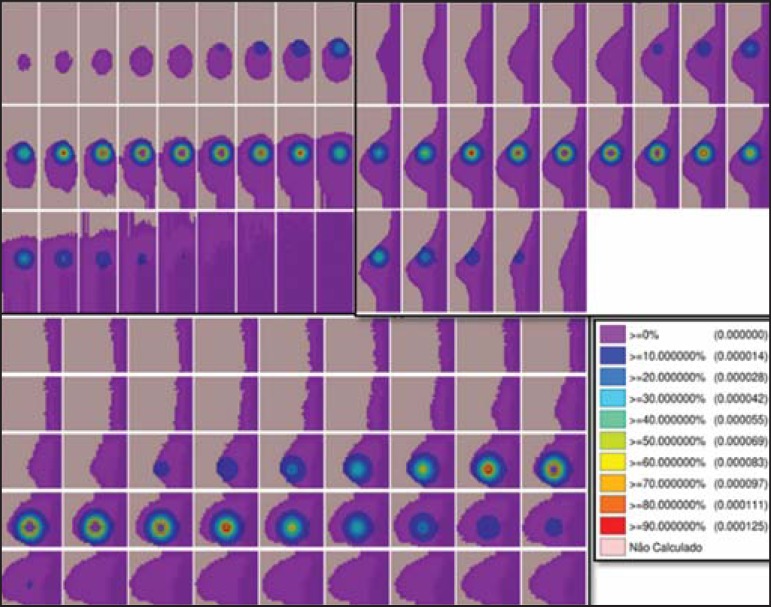
Spatial distribution of normalized dose rate in relation to the activity, shown in lateral, sagittal, and axial slices, induced by implantation of a balloon with a homogeneously distributed source of ^99m^Tc.

Figures 1 and 2 show the results of the temporary implant protocol simulations with a ^99m^Tc-filled interstitial balloon and with a ^192^Ir-filled segment, mimicking a balloon brachytherapy booster.

### HDR **^192^** Ir-filled temporary balloon implant

Figure 1 illustrates the spatial distribution of a normalized dose in relation to the total activity of the ^192^Ir-filled balloon. Combined lateral, sagittal, and axial sections with dose values higher than 10% are shown. The dose was administered by the discrete ^192^Ir source, inserted in the intracavitary balloon. In the simulation conditions imposed, the maximum normalized dose rate reached 0.499 cGy.h^-1^.mCi^-1^. According to the distribution of doses in Figure 1, it is possible to predict the dose away from the balloon surface.

### **^99m^** Tc-filled temporary balloon implant

Figure 2 illustrates the spatial distribution of a normalized dose in relation to the total activity of the ^99m^Tc-filled balloon. Combined lateral, sagittal, and axial sections with dose values higher than 10% are shown. The dose was administered from an aqueous solution-distributed, homogeneous Na(TcO_4_)^-^ source. The simulated balloon measured 16 mm in diameter. In this case, the maximum normalized dose rate reached 0.428 cGy.h^-1^.mCi^-1^.

Table 2 shows the dosimetric values in the simulations of both protocols. The maximum dose rate (MDR) was multiplied by the dose percentage (DF) factor in relation to the reference point (RP), that is, at 8-10 mm from the balloon surface; then by the total activity at the source and by the accumulation factor (AF) of the exposure time (T), thus producing the accumulated dose at the RP. With the definition of the number of fractions (FR), the accumulated dose for the protocol at the RP was found.

**Table 2:** Dosimetric intercomparison of radiotherapy boost in the breast, in situations of increased complementary dose in the tumor bed.

Balloon brachytherapy protocol	DR_100_	DF at the RP (%)	Activity (mCi)	T(FR) (h)	Nuclide	Half-life (h)	AF	(D_F_)D_T_ (G_y_)
^99m^Tc solution*	0.428	25	1000	24	^99m^Tc	6.01	8.126	10.14
HDR ^192^Ir^†^	0.499	30	10000	0.4 (2)	^192^Ir	1771.92	0.400	5.14 (10.28)

* Interstitial balloon, measuring 16 mm in diameter, implanted postoperatively in the tumor resection cavity, with external catheter. Balloon filled with homogeneous Na(TcO_4_)– radioactive solution.

† Afterloading interstitial brachytherapy using positioned catheters, insertion of HDR Ir^192^ linear metallic cylindrical segment measuring 1 × 5 mm at the center of the 40-mm diameter water-filled balloon.

DR_100_, dose rate (in cGy.h^–1^.mCi^–1^) at 100%, obtained in the simulation investigated in all voxels; RP, reference point for comparative evaluation of the absorbed dose, at 8 mm from the balloon surface; DF, dose factor at the RP, representing the maximum dose percentage estimated at the position starting at the spatial distribution of the dose generated in the computer simulation; T(FR), the fraction (fractional situation), adjusted by the time per fraction T (hours); AF, accumulation factor (1 – exp(λT))/λ, where λ is the decay constant and T is the exposure time); D_F_, fractionated dose; D_T_, total accumulated dose where D_F_ was multiplied by FR.

The ^99m^Tc-filled balloon had 1 Ci of Na(TcO_4_)^-^ and an RP at a distance of 10 mm from the balloon surface, within 25% of the MDR. For a T of 24 hours, the AF was 0.812. The HDR ^192^Ir-filled balloon, on the other hand, had 10 Ci of ^192^Ir, at the same RP, and the DF was therefore 30% of the MDR. A T of 0.4 hours produced an AF of 0.4. Under these conditions, the accumulated doses at the RP were 10.14 Gy for the ^99m^Tc-filled balloon and 5.14 Gy for a single fraction of the HDR ^192^Ir-filled balloon, which will produce an equivalent dose of 10.28 Gy in two sections.

## DISCUSSION

In this study, we have shown that a boost of 10 Gy, complementary to teletherapy, can be produced in both protocols, at the chosen reference positions. There were no statistical differences between the ^99m^Tc-filled and ^192^Ir-filled balloons, in terms of the accumulated doses, considering the 5% variation given by the simulation. In this case, ^99m^Tc-filled and ^192^Ir-filled balloons, in the target conditions of activity and exposure, produced equivalent absorbed doses, under the proposed conditions.

The dosimetric intercomparison between the techniques was differentiated by the spatial distribution of the source and by the diameter of the balloon. This simulated condition refers to resected, in situ tumors in the initial stages. Therefore, the partial volume of the resected tumor is filled by the balloon, which may be inflated, occupying the cavity and expanding the adjacent tissue. The dose in the tumor bed, in this case, was represented by the dose in the voxels at the edges of the balloon.

The ^99m^Tc-filled balloon implant may be recommended for stage T1 and T2 tumors with a 1 cm margin, in the usual post-lumpectomy cavity^(7-12)^. Radiation attenuation due to the radial distance is greater for the ^99m^Tc-filled balloon than for the ^192I^r-filled balloon, because ^99m^Tc has lower energy emissions than does ^192^Ir^(30,32)^. Consequently, the radial dose profile will become more restricted towards the surgical cavity, as may be observed in Figure 2 (^99m^Tc), in comparison to Figure 1 (^192^Ir). Therefore, the lungs and heart are expected to receive smaller doses with the ^99m^Tc-filled balloon than with the ^192^Ir-filled balloon, thus exerting fewer deleterious effects on those healthy organs. At the tumor bed, the spatial distribution of the dose from the HDR ^192^Ir-filled balloon showed a MDR per unit of activity equivalent to that of interstitial brachytherapy with the ^99m^Tc-filled balloon.

The MDR per unit of activity are comparable. However, because the HDR maintains the ^192^Ir activity at a level 3.5-5.0 times greater than that of the ^99m^Tc, the dose rate will increase in the same proportion. Therefore, at 8 mm from the balloon, the dose rate for 1.0 Ci of ^99m^Tc will be 1.07 Gy.h^-1^, reaching 0.13 Gy.h^-1^ in 18 hours, whereas the dose rate for 10.0 Ci of ^192^Ir remains practically constant for a period of 25 minutes, equal to 12.83 Gy.h^-1^ (12 times greater than the initial dose rate for the ^99m^Tc-filled balloon). The lower rate, however, may reduce the deleterious effects on healthy tissues, offering better repair of the inherent sublethal damage to normal cells.

Generators of ^99^Mo/^99m^Tc are normally distributed with 2.0 Ci. Therefore, the ^99m^Tc-filled balloon implant technique could be immediately incorporated without additional costs, with the support from nuclear medicine facilities. The necessary radiation safety procedures for the use of high activity (7-10 Ci) ^192^Ir sources, which have a half-life of 73.8 days, should be stricter than those employed for ^99m^Tc generators. Consequently, greater management complexity is expected from the use of ^192^Ir, considering acquisition, transportation, and substitution of the cylindrical radiation source every three months at the centers that offer HDR brachytherapy, particularly due to the need to import the devices and employ specialized teams to replace their sources.

A normal protocol for conventional radiotherapy involves the application of 25 sessions of 1.8-2.0 Gy per day, with two parallel opposed fields of 6 MV, for example, 5 days a week, for 45-60 days, with accumulated doses of 50 Gy covering the mammary gland tissue^(5)^. For boosts in HDR ^192^Ir brachytherapy, maximum exposure is achieved in sessions of 20-25 minutes, compared with 24 hours for the ^99m^Tc-filled balloon technique. Longer periods of exposure translate to greater patient discomfort. A 10-Gy boost dose at the tumor bed using the ^99m^Tc-filled balloon technique requires 24 hours of exposure, plausibly generating such discomfort. The technique, however, may be applied postoperatively in a single fraction, the balloon being implanted immediately after local excision and exposure taking place during the recovery period. If the activity injected is doubled, the exposure time is reduced by half, or a multifraction protocol involving 4-6 hours of exposure per day, for 3 to 4 days, may be implemented, in which the radioactive liquid is replaced with water during the periods between exposures, and the radioactive solution is injected with constant activity at every application. The prescribed dose for the tumor bed should be previously defined, in order to establish the levels of activity and exposure.

In well-established studies, with long-term follow-up, conducted in Europe and the United States, brachytherapy with a HDR ^192^Ir-filled balloon has been proven to be a safe and efficient method, with low rates of local recurrence^(13-17)^. However, its application is limited because of the complexity of the procedure and the long (73.8 day) half-life of high-activity ^192^Ir sources. In addition, there are contraindications that can interfere with dosage planning^(34)^: insufficient distance between the tumor and the skin; and an extensive cavity. Its use is not indicated when the tumor cavity is in an area of tissue that cannot be sufficiently covered^(35)^. Undesirable anatomic surgical conditions and ineligible histological conditions can also preclude the application of the balloon technique^(17)^. With a smaller balloon and a nuclide that provides greater dose attenuation, ineligible clinical conditions, due to inappropriate or compromised anatomy with reduced coverage tissue mass, may become less relevant.

In general, the ^99m^Tc-filled balloon technique is accessible, has a shorter learning curve, presents less complex dosimetry, and is widely available, considering the ^99^Mo/^99m^Tc generators at nuclear medicine facilities in Brazil.

## CONCLUSION

Temporary ^99m^Tc-filled balloon implants could represent an attractive option for adjuvant radiotherapy in breast cancer. The technique supplies an adequate boost dose, with spatial dose distribution contained within the tumor bed surroundings, and its use is justified by its availability and economic viability.
